# Synthetic and Spectroscopic Characterization of Organotin(IV) Complexes of Biologically Active Schiff Bases Derived from Sulpha Drugs

**DOI:** 10.1155/S1565363303000244

**Published:** 2003

**Authors:** M. K. Gupta, Har Lal Singh, S. Varshney, A. K. Varshney

**Affiliations:** Department of Chemistry University of Rajasthan Jaipur 302004 India

## Abstract

A number of diorganotin(IV) complexes with Schiffbase have been synthesized and characterized by elemental analysis, conductance measurements, molecular weight determinations, infrared, electronic and multinuclear magnetic resonance (^1^H, ^13^C and ^119^Sn NMR) spectral data. The molar conductivity data shows non-electrolytic nature of complexes. The bidentate nature of the ligands is inferred from IR and NMR spectral studies. The antimicrobial activities of the ligands and their tin complexes have been screened *in vitro against the organism Escherichia coli; Staphylococus aureus, Prouteus mirabilis, Bacillus thurengiensis, Penicillium co.,sogenum, Aspergillus niger and Fusarium oxysporum*.


### Synthetic and Spectroscopic Characterization of Organotin(IV) Complexes of Biologically Active Schiff Bases Derived from Sulpha Drugs

M. K. Gupta, Har Lal Singh, S. Varshney and A.K. Varshney*

*Department of Chemistry, University of Rajasthan, Jaipur-302004, India.*
*E-mail: anilakv123@rediffmail.com*

### ABSTRACT

A number of diorganotin(IV) complexes with Schiff base have been synthesized and characterized by elemental analysis, conductance measurements, molecular weight determinations, infrared, electronic and multinuclear magnetic resonance ($^{1}H$, $^{13}C$ and $^{119}Sn$ NMR) spectral data. The molar conductivity data shows non-electrolytic nature of complexes. The bidentate nature of the ligands is inferred from IR and NMR spectral studies. The antimicrobial activities of the ligands and their tin complexes have been screened *in vitro* against the organism *Escherichia coli*, *Staphylococus aureus, Prouteus mirabilis, Bacillus thurengiensis, Penicillium crysogenum, Aspergillus niger and Fusarium oxysporum*.

**Keywords:** Dibutyltin oxide, heterocyclic aldehydes/ketones, organotin(IV) complexes, spectral studies, microorganism.

### INTRODUCTION

Organotin compounds show a large spectrum of biological activities. In recent years, several investigators to test their antitumour activity /1,2/ have been carried out. It has been observed that several organotin complexes are effective antifouling, /3/ antmicrobial /4/ and antiviral agents, therefore, much attention has been paid to their implications for antioncogenesis /5/. They are also used commercially as bactericides, fungicides, acaricides and industrial and agriculture biocides /6,7/. The pronounced biological activity of the metal complexes of Schiff bases derived from sulpha drugs has led to considerable interest in their coordination chemistry. The condensation products of sulpha drugs with aldehydes and ketones are biologically active and also have good complexing ability; their activity increases on complexation with metal ion /8-11/. Keeping this in view, it was considered worthwhile to synthesize organotin complexes of some stereochemical as well as biological importance. During the course of the present investigations, an attempt has been made to synthesize organotin complexes by interacting $Bu_2SnO$ and Schiff bases derived by condensation of heterocyclic aldehydes with various sulpha drugs. The structures of the ligands are shown in Fig. 1.

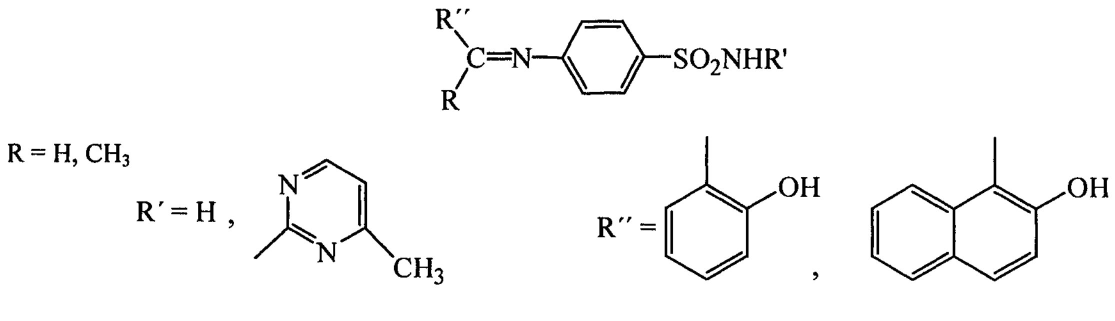

**Fig. 1:** Schiff bases used as ligands in this work

### MATERIALS AND METHODS

Chemicals and solvents used were dried and purified by standard methods /12/ and moisture was excluded from the glass apparatus using $CaCl_2$ drying tubes. Melting points were determined in open capillaries and are uncorrected.

### Preparation of the Schiff Bases

The Schiff bases were synthesized by the condensation of 2-hydroxy-1-naphthaldehyde, o-hydroxyacetophenone and salicylaldehyde with sulpha drugs, *viz.* sulphanilamide and sulphamerazine, in 1:1 molar ratio using ethanol as the reaction medium. The solution was refluxed on a water bath for 4-5 h and then allowed to cool at room temperature. The crystalline solids were separated out and purified by recrystallization from the same solvent. The main characteristics and analytical data are recorded in Table-1.

### Preparation of Organotin(IV) Complexes

Organotin(IV) oxide was added to the calculated amount of Schiff base in a 1:2 molar ratio in dry benzene as reaction medium. The contents were refluxed on a fractionating column for about 24 hours. The water liberated in the reaction was removed azeotropically with benzene. On completion of the reaction, the resulting products were rendered free from solvent and then washed repeatedly with dry cyclohexane. The crystalline solids were separated out and purified by recrystallization from the same solvent. The products so formed were finally dried *in vacuum* at 40±5 °C for 2-3 hours. The purity of the compounds was checked by TLC using silica gel-G as adsorbent. Their physical properties and analytical data are recorded in Table 2.

### Analytical Methods and Spectral Measurements

Tin was estimated gravimetrically as $SnO_2$. Nitrogen and sulphur were estimated by Kjeldahl's and Messenger's methods, respectively /13/. Molar conductance measurements were made in anhydrous DMF at 36±1 °C using a Systronics conductivity bride-305. Molecular weight determinations were carried out by the Rast camphor method. The electronic spectra were recorded in methanol on a Toshniwal spectrophotometer. Infrared spectra were obtained on a Nicolet Magna 550 FTIR spectrophotometer as Nujol mulls as CsI window or KBr discs. $^1H$ NMR spectra were recorded on a Perkin-Elmer RB-12 spectrometer in $CDCl_3$ using TMS as internal standard at 90 MHz. $^{13}C$ and $^{119}Sn$ NMR spectra were recorded on a 90 MHz Jeol Fx-90 Q NMR spectrometer in $CHCl_3$ using TMS as an internal standard and TMT as an external standard at 22.8 and 22.7 MHz, respectively.

### Antibacterial Test

*In vitro* antibacterial activity of the ligands and the complexes was tested using the paper disc diffusion methods /14/ at a concentration of 100 ppm. Streptomycin was used as reference compound for antibacterial activities. *E. coli*, *S. aureus*, *Bacillus thurengiensis* and *P. mirabilis* were used as the test organisms.

### Antifungal Activity

In the radial growth method, the medium (potato, dextrose, agar and distilled water) and fungi were grown at 28±2 °C. The compounds were mixed in 50, 100, 200 ppm concentrations in the medium. The linear growth of the fungus was obtained measuring the colony diameter after 9 h and the average of three replicates of growth in mm was considered. The amount of growth inhibition was calculated by the equation given by Vincent /15/:

$$\% \text{ inhibition} = (C-T) \times 100/C$$

C= diameter of fungus colony in control plate

T= diameter of fungus colony in test plate

### RESULTS AND DISCUSSION

The reaction of organotin(IV) oxides with the Schiff bases of sulpha drugs with the elimination of water, which was removed azeotropically with benzene:

$$Bu_2SnO + 2NOH \longrightarrow Bu_2Sn(NO)_2 + H_2O$$

where NOH represents the Schiff bases of Sulpha Drugs

The above reactions are quite facile and could be completed in 22-24 hours of refluxing in benzene and removing the liberated water azeotropically. All these newly synthesized complexes are coloured solids and are soluble in DMF, DMSO and partia[illegible] soluble in common organic solvents. The conductance of these complexes has been recorded in DMF at room temperature in the range 8-20 $ohm^{-1}cm^{2}\ mol^{-1}$, suggesting their non-electrolytic nature. The molecular weights of the complexes de[illegible]mined by the Rast camphor method correspond with formula weight, indicating their monomeric nature. The physical characteristics of these complexes are given in Table 2

### Electronic Spectra

The UV and visible spectra of Schiff bases exhibit two intense absorption maxima at 246 and 390 nm. The band at 246 nm is assignable to π-π* (benzenoid) transitions which are shifted towards the lower energy region (266 nm) in the spectra of tin complexes. Such a bathochromic shift may be due to an increase in the availability of the lone pair of electrons on the auxochromic oxygen, as the intramolecular H-bonding initially present in the ligand ceases after the deprotonation of the OH group on complexation. The bands at 390 nm in the spectra of the ligands are due to n-π* transition within the >C=N chromphore. An appreciable blue shift in these bands is observed in the spectra of the tin complexes and it may be attributed to the coordination of azomethine nitrogen to the tin atom.

### Infrared Spectra

In the IR spectra of the ligands, a strong band in the region $1610\pm10\ cm^{-1}$ assignable to $\nu$(C=N) /16/ is shifted to the lower frequency side (~15 $cm^{-1}$) in the spectra of the tin complexes, indicating coordination through the azomethine nitrogen to the tin atom /17,18/. The spectra of these derivatives do not show any bands in the 2770 $cm^{-1}$ regions which could be assigned to the hydrogen bonded $\nu$(OH) vibration originally present in the ligands. This indicates complexation with the tin atom. Medium to strong intensity bands appear at ~1260 $cm^{-1}$ and may be assigned to the phenolic C-O stretching mode. This band is slightly shifted to the higher frequency side (~1285 $cm^{-1}$) in the spectra of the tin complexes, showing chelation of the phenolic oxygen to the tin atom.

The appearance of new strong to medium intensity bands is observed at ~550 $cm^{-1}$ and ~600 $cm^{-1}$, which may be assigned to the symmetric and asymmetric mode of $\nu$(Sn-C) /19/, stretching vibrations in the spectra of tin complexes and also two bands at $530 \pm 10$ and $410\pm 12\ cm^{-1}$ may be assigned to $\nu$(Sn-O) /20/ and $\nu$(Sn←N) /21/ vibrations, respectively, indicating the participation of azomethine and phenolic oxygen in complexation.

### $^{1}$H NMR Spectra

The $^{1}$H NMR data of ligands and their organotin(IV) complexes have been recorded in $CDCl_3$ (Table 3). In the $^{1}$H NMR spectra the ligands show a signal at δ 12.35-13.16 ppm for the hydrogen bonded phenolic protons. These signals completely disappear in the complexes, indicates that the chelation of phenolic oxygen to the tin atom. In the case of the ligands the proton signal for the methyl protons [-C($CH_3$)=N] and azomethine protons [-CH=N] in the region δ ~1.88 ppm and δ ~8.95 ppm, respectively, shifts downfield in the spectra of corresponding tin complexes (δ ~0.10 ppm) on account of its deshielding, which is attributed to the donation of the lone pair of electrons by the azomethine nitrogen to the tin atom. The ligand shows a complex multiplet in the region δ ~7.90-6.80 ppm for the aromatic protons which remains at almost the same position in the spectra of the organotin(IV) complexes. The complexes, however, show additional signals at δ ~0.75 – 1.95 ppm owing to the protons of the butyl group.

### $^{13}C$ NMR Spectra

The $^{13}C$ NMR spectral data for salicylaldehyde sulphanilamide, 2-hydroxy-1-naphthaldehyde sulphanilamide, o-hydroxyacetophenone sulphanilamide and its corresponding tin complexes are reported in Table 4. The shifting in the position of resonance of carbon attached to OH group suggests the bonding of oxygen to the tin atom. Further, the shifting of azomethine (>C=N-) carbon signal in the spectra of complexes as compared to the ligands clearly indicates that the azomethine moiety has been involved in coordination. The carbon of the butyl group is observed at (δ ~26.5, ~27.4, ~26.2, ~14.0 ppm) position comparable to other similar compounds. The R group attached to tin displays resonance for chemically equivalent carbon; however, the butyl compounds display three resonance. The tin-carbon coupling $^{n}J(^{119}Sn-^{13}C)$ values of n=1, 920 Hz; n=2, 40.5 Hz; and n= 3, 125.8 Hz indicate the six coordinate around tin in such organotin(IV) complexes.

### $^{119}Sn$ NMR Spectra

The $Bu_2Sn(IV)$ complexes give sharp signals at ~δ –350.2 ppm, in $^{119}Sn$ NMR spectra, which strongly supports the six coordination around tin in a distorted octahedral geometry. Values /22-24/ for similar six coordinated $Bu_2Sn(IV)$ complexes have been reported in the range of δ –265 to -365 ppm.

On the basis of the observed spectral evidence, the following tentative structures with (probably distorted) octahedral geometry can be proposed:

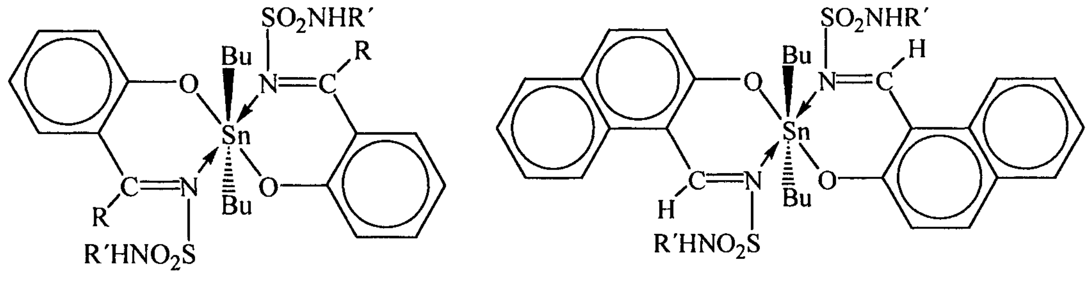

**Fig. 2:** Geometry of the Organotin(IV) Complexes

### Antimicrobial Results

All the complexes were tested against gram positive and gram negative bacteria. The results listed in Table 5 show that all the complexes were active against gram positive bacteria while less active against *E. coli*, which is gram negative.

The results further show that complexes having hydroxy naphthalene nucleus were more active then salicylaldehyde or hydroxyacetophenone Schiff base counterparts. This is in accordance with the well established view that the naphthalene nucleus possessing –OH group increases the activity of a compound /25/. Further, the tin complexes are more active as compared to the ligands, which indicates that metallation increases the activity /26,27/. All the compounds tested (Table 6) were found to be highly active against *penicillium crysogenum, Aspergillus niger* and *Fusarium oxysporum*. The activity was highly marked in case of *A. niger*, the growth of which was maximally inhibited at 100 ppm concentration is the some compounds. The high activity of these complexes may also be explained as the basis of the fineness of their particles, which is an important factor for the biological activity. The above studies clearly indicate that the tin complexes synthesized in the present studies are highly active against all these pathogens.

### ACKNOWLEDGEMENTS

A.K.V. is thankful to UGC for financial assistance as a minor project. One of the authors (H. L. Singh) wishes to thank the Council of Scientific Industrial Research, New Delhi for financial assistance. The authors are thankful to the Head, Department of Chemistry, University of Rajasthan, Jaipur, for providing laboratory facilities.

### REFERENCES

1. M. Gielen, *Appl. Organomet. Chem.*, **16**, 481 (2002).
2. D.V. Dick, W. Rudolph, M. Gielen, V. Wingerden, E. Kyra and N. Kees, *Metal-Based Drugs*, **5**, 179 (1998).
3. D. C. Cinito Roberto and D. C. Giorgio, *Acquo Aria*, **9**, 993 (1993); *Chem. Abstr.*, **120**, 156632 (1994).
4. M. Nath and S. Goyal, *Metal Based Drugs*, **2**, 297 (1995); *Chem. Abstr.*, **124**, 79429 (1996).
5. V. Narayanan, M. Nasr and K.D. Paull, in: *Tin-Based Anti-Tumor Drugs*, M. Gielen (ed.), Springer: Berlin, 1990; p.201.
6. W. T. Piver, *Environ. Health Perspect*, **4**, 61 (1973).
7. G. M. J. Vande Kerk, In *Organotin Compounds*, J. J. Zuckerman (ed.), American Chemical Society: Washington, 1968; p.1.
8. G. D. Tiwari and M. N. Mishra, *Curr. Sci.*, **49**, 8 (1980).
9. K. Lal and R. K. Shula, *J. Indian Chem. Soc.*, **LVIII**, 115 (1981).
10. P. Jain and K. K. Chaturvedi, *J. Inorg. Nucl. Chem.*, **39**, 109 (1977).

11. H. L. Singh, S. Varshney and A. K. Varshney, *Appl. Organomet. Chem.*, **13**, 637 (1999).
12. D.D. Perrin, W.L.F. Armarego and D.L. Perrin, *Purification of Laboratory Chemicals*, 3rd edn. Pergamon : London, 1988.
13. B.S. Furniss, A.J. Hannaforal, P.W.G. Smith and A.R. Tatchell, *Vogol's Text Book of Practical Organic Chemistry*, 5th edn. Longman, 1989.
14. D. Lui and Kwasniewska, *Bull.Environm.Toxicol.*,**27**, 289 (1981).
15. J. M. Vincent, Farmers. *Bull U. S. D. A. Inhibitor Nature*, **159**, 850 (1959).
16. M. Vazquez, M. R. Bermejo, M. Fondo, A. Garcia-Deibe, A. M. Gonzalez and R. Pedrido, *Appl. Organomet Chem.*, **16**, 465 (2002)
17. A. K. Varshney and J. P. Tondon, *Proc. Ind. Acad. Sci. (Chem. Sci)*, **94**, 509 (1985).
18. H. L. Singh, M. Sharma, and A.K. Varshney, *Synth. React. Met.-Org. Chem.*, **29**, 817 (1999).
19. N. S. Biradar and V. H. Kulkarni, *J. Inorg, Nucl. Chem.*, **33**, 2451(1971).
20. W. D. Honnick and J. J. Zuckerman, *J. Organomet. Chem.*, **178**, 133 (1979).
21. P. Jain and and K. K. Chaturvedi, *J. Indian Chem. Soc.*, **52**, 805 (1975).
22. M.S. Singh, David, Raju, Kiran Tawade and A.K. Singh, *Main Group Met. Chem.*, **21**, 489 (1998).
23. T. P. Lockhart and W. F. Manders, *J. Am. Chem. Soc.*, **109**, 7015 (1987).
24. C. Pettinari, M. Pellei, M. Miliani, A. Cingolani, A. Cassetta, L. Barba, A. Pifferi and E. Rivarola, *J. Organomet.Chem.*, **553**, 345 (1998).
25. C. Pettinari, F. Marchetti, A. Cingolani and S. Bartolani, *Polyhedron*, **15**, 1263 (1996).
26. J. G. Horsfall, "Fungicides and their action" in: *Chronica Botanica*, Walthan, Massachusetts, 1945.
27. H. L. Singh, B. Khungar, U. D. Tripaathi and A. K. Varshney, *Main Group Met. Chem.*, **24**, 5 (2001).
28. M. Nath and S. Goyal, *Bull. Chem. Soc. Jpn.*, **69**, 605 (1996).

